# Gender Differences of Arterial Stiffness and Arterial Age in Smokers

**DOI:** 10.3390/ijerph14060565

**Published:** 2017-05-26

**Authors:** Ioana Mozos, Jean Paul Maidana, Dana Stoian, Milan Stehlik

**Affiliations:** 1 Department of Functional Sciences, “Victor Babes” University of Medicine and Pharmacy, 300173 Timisoara, Romania; ioanamozos@yahoo.de; 2 Center for Translational Research and Systems Medicine, “Victor Babes” University of Medicine and Pharmacy, 300173 Timisoara, Romania; 3 Institute of Statistics, University of Valparaiso, 234000 Valparaíso, Chile; jean.maidana@postgrado.uv.cl (J.P.M.); mlnstehlik@gmail.com (M.S.); 4 2nd Department of Internal Medicine, “Victor Babes” University of Medicine and Pharmacy, 300723 Timisoara, Romania; 5 Department of Applied Statistics, Johannes Kepler University, 4040 Linz, Austria; 6 Linz Institute of Technology, Johannes Kepler University, 4040 Linz, Austria

**Keywords:** smoking, arterial stiffness, pulse wave velocity, arterial age, blood pressure, male, female

## Abstract

The present study aimed to find gender differences for arterial stiffness and arterial aging in smokers. A total of 147 smokers (71 male and 76 female, matched for age) were explored using an Arteriograph in a cross-sectional survey. Pulse wave velocity (PWV), arterial age (AA), brachial and aortic augmentation index (AixBrach, AixAo), and blood pressure variables were assessed. Data about smoking intensity, such as the number of cigarettes smoked daily, smoking period, and smoking pack years (SPY) were used. No significant differences were found for PWV, AA, AixBrach and AixAo. Significant correlations were found between SPY and PWV, augmentation indices, and AA, respectively. The cut-off values for SPY were higher for an increased arterial stiffness in male compared to female smokers (18.5 and 7.5 pack year, respectively). SPY is significantly associated with an increased arterial stiffness in smokers regardless of gender, and with an increased SBPAo only in female smokers. The results of our study indicate gender differences for arterial stiffness and arterial age in smokers.

## 1. Introduction

Public health programs and legislation significantly decreased the prevalence of tobacco smoking and secondhand tobacco smoke exposure in several countries [1]. Smoking is a leading cause of premature mortality and a known modifiable environmental cardiovascular risk factor, previously reported to be associated with endothelial dysfunction, arterial stiffness, and early arterial aging [2,3]. Both active and passive cigarette smoking were associated with endothelial dysfunction in a dose-dependent manner, probably related to free radicals of cigarette smoke able to inactivate nitric oxide (NO) [4,5]. Even the smoking of one cigarette impaired endothelial function in a study including healthy college students [6].

Arterial stiffness is a marker of arteriosclerosis and subclinical atherosclerosis, and provides information beyond standard cardiovascular risk factors in the prediction of future cardiovascular events [7]. Current European guidelines acknowledge the good cardiovascular predictive value, reproducibility, and cost-effectiveness of pulse wave velocity (PWV) [8]. Arterial stiffness may be assessed using the pulse wave velocity or the augmentation index—a wave reflection parameter and an indirect marker of arterial stiffening [9]. Arterial age is the chronological age of a person with all risk factors at normal levels and the same 10-year predicted risk [10]. Changes in arterial stiffness may be due to vascular remodeling, affecting arterial wall fibers, heart rate, endothelial function, inflammation, oxidative stress, turnover in the extracellular matrix of the vessel wall, sympathetic tone, and genetic polymorphism [11,12].

All-cause and cardiovascular mortality are higher in men than in women, but there is a higher increase of cardiovascular mortality in women with age, especially after menopause, due to estrogen deficiency [13]. Gender differences in smoking and smoking withdrawal vary across countries and different cultures, according to several socio-economic and individual factors [14,15].

In Romania, the percentage of declared smokers was 30% in 2010, easily exceeding the European average in percentage of smokers, and the average number of cigarettes smoked daily was 15 [1]. However, it is not clear whether gender has an influence on vascular changes caused by smoking.

It was the aim of the present study to explore gender differences of arterial stiffness, endothelial function, and arterial aging in smokers.

## 2. Materials and Methods

Study population: A total of 147 consecutive volunteer smokers (71 male and 76 female) recruited from a general practitioner’s office were explored using an Arteriograph in a cross-sectional survey. Data about smoking habits were collected. All clinical and biological parameters were evaluated on the day of Arteriograph examination. The investigations conformed to the principles outlined in the Declaration of Helsinki, and were approved by the “Victor Babes” University (2743/9 March 2016). Written informed consent was obtained from each study participant. The purpose and procedures of the study were explained to each participant.

Exclusion criteria: patients with atrial fibrillation, diabetes mellitus, history of coronary heart disease, myocardial infarction, stroke, transient ischemic attack or peripheral arterial disease, and therapy with drugs known to influence arterial stiffness (e.g., statins) were excluded.

Arteriograph: An Arteriograph—an oscillometric, noninvasive, validated, operator-independent device (TensioMed Ltd., Budapest, Hungary)—was used to assess central and peripheral blood pressure variables, pulse wave velocity (PWV), augmentation indices (brachial: AixBrach and aortic: AixAo), and arterial age (AA) in each study participant. PWV is a measure of arterial stiffness, and assesses the velocity of the movement of the pulse wave generated during ejection of the left ventricle, along the arterial tree. As the pulse wave travels from the aortic root to the peripheral vessels, a first systolic direct wave results, and due to diameter changes and bifurcations, a second reflected wave occurs. The augmentation index was automatically calculated from the blood pressure waveform as the difference between the amplitude of the reflected (late systolic) and direct (first systolic) wave, divided by the pulse pressure and multiplied by 100. The pulse pressure is the difference between systolic and diastolic blood pressure. Arterial age is a measurement of aortic PWV, which depends on age and was automatically calculated by the Arteriograph.

The methodology and the significance of the variables were previously described [16]. Recordings were made according to the recommendations for the standardization of subject conditions [8,17]. Two measurements were performed in each participant by one of the authors of the present paper, blinded for the smoking variables, diagnosis, therapy, and family cardiovascular history of the patients in order to examine the reliability of the measurements. A cut-off value of 9.7 m/s was recommended by the manufacturer of the device for increased PWV.

Smoking variables: The study participants were interviewed to determine smoking status and data about smoking habits (self-reported): the number of cigarettes smoked daily and smoking period. Smoking pack years (SPY) was calculated as an expression of lifetime tobacco exposure. One “pack year” is 20 cigarettes smoked/day for 1 year [18,19].

Other variables: Data about cardiovascular family history, diagnosis, and therapy were also collected from medical records.

Statistical analysis: Categorical data are given as numbers and percentages; continuous data as means ± standard deviation. Standard deviations were calculated from the unbiased estimator of the variance. Binary logistic regression analysis with a single categorical predictor, Bravais-Pearson correlation coefficient, Wilcoxon test, ANOVA, and receiver-operating characteristic (ROC) curve analysis were used as statistical methods. Analyses were performed using Excel 2016, IBM SPSS Statistics Free trial, the STATS package from The Comprehensive R Archive Network (R for the Wilcoxon test), and a self-programmed algorithm with Python) (ANOVA). Wilcoxon test and ANOVA were used to report significant differences between male and female smokers. Logistic regression analysis and Bravais–Pearson correlation assessed the relationship between SPY and variables obtained during oscillometric measurements. ROC curve analysis allowed a complete sensitivity/specificity report of SPY for an increased AixBrach, systolic blood pressure in the aorta (SBPAo), and PWV.

The power analysis conducted to determine the sample size needed for the present study showed a minimum sample size required for Bravais–Pearson correlations of 29 participants (desired statistical power: 0.8; correlation coefficient r: 0.5; significance level: 0.05). The required sample size for an area under the curve (AUC) of 0.8, 95% confidence interval, 80% power, and a marginal error of 0.12 was 61.

## 3. Results

The characteristics of the male and female study population and the obtained variables are included in Table 1.

### 3.1. Significant Differences

Men had significantly higher values for SPY, number of cigarettes smoked/day, BMI, and blood pressure values compared to female smokers. Brachial augmentation index was more severely impaired in female compared to male smokers, but the differences were statistically significant only after adjusting for heart rate (Table 1). No significant differences were found for PWV, AA, and AixAo between male and female smokers. AixAo was significantly higher in female smokers only after adjusting for heart rate.

### 3.2. Correlations

More important correlations were found between SPY and arterial age, AixAo and AixBrach, respectively (Table 2), in male compared to female smokers.

### 3.3. Logistic Regression Analysis

Binary logistic regression analysis was performed separately for male and female smokers. SPY was associated with an increased arterial stiffness in both male and female patients (Table 3).

### 3.4. Receiver-Operating Characteristic (ROC) Curve Analysis

ROC curve analysis revealed SPY as a diagnostic test for AixBrach ˃ 10% and increased arterial stiffness (PWV ˃ 9.7 m/s), regardless of gender. SPY was also a significant diagnostic tool for an increased systolic blood pressure in the aorta (˃130 mmHg) in female smokers. The cut-off values for SPY—related to an increased arterial stiffness—were higher in male compared to female smokers (Table 4).

## 4. Discussion

The present study shows gender differences for the vascular consequences of smoking—especially for arterial stiffness. SPY was significantly associated with an increased arterial stiffness regardless of gender, but a lower exposure to cigarette smoke is needed in order to increase arterial stiffness in female compared to male smokers. SPY affects arterial age more severely in male smokers.

Smoking has been defined as a chemical toxicosis, and several harmful compounds were found in tobacco, such as nicotine, tar, thiocyanate, and carbon monoxide [20,21]. Mainstream smoke (inhaled by the smoker directly), and side-stream smoke emanating from the burning end of the cigarette—a major component of secondhand tobacco exposure—should be considered [22]. Nicotine is an alkaloid found in the leaves of tobacco plant which stimulates the sympathetic system and the release of thromboxane A2, increasing heart rate, blood pressure, and cardiac output, and is responsible for the addictive effect of tobacco [11,20,21]. Chronic smoking was associated with increased reactive oxygen species generation in mononuclear and polymorphonuclear cells, impairing nitric oxide bioavailability, dysfunctional NO synthase, increased consumption of NO, hypertension, activation and aggregation of platelets, changes in hemostatic components and blood lipids, a decrease in arterial collagen content, acceleration of the atherosclerotic process, and thrombosis, increasing the level of proinflammatory cytokines [5,9,11,23]. Subjects with more advanced vascular damage are more vulnerable to the acute effects of cigarette smoking [24]. Females were found to have higher mean values of total cholesterol, total lipids and phospholipids, and lower high-density lipoprotein cholesterol (HDL), and smokers, as well, higher values for total cholesterol and lipids [25]. Higher levels of cotinine—a marker of secondhand smoke exposure—was positively associated with PWV and brachial pulse pressure, demonstrating the detrimental vascular effect of passive smoking, probably related to vasoconstriction mediated by nicotine [26].

Previous studies reported higher aortic systolic blood pressure and augmentation index in chronic smokers compared to nonsmokers—regardless of gender—and increase of blood pressure, augmentation index, and pulse wave velocity after smoking of one cigarette, regardless of smoking status [27]. The increase in central systolic and pulse blood pressure in smokers is attributable to a reflected pressure wave increased in amplitude, duration, and velocity, which enhances arterial wall stress, potentiates the development of atherosclerosis, and increases left ventricular mass and oxygen demand [11]. Deterioration of arterial stiffness and systemic endothelial activation in smokers is mediated by the upregulation of cyclooxygenases 1 and 2, considering that aspirin prevented the smoking-induced increase of PWV [28]. The acute detrimental effect of cigarette or cigar smoking—even passive smoking—includes increase of both brachial and central blood pressure and arterial tone, as measured by aortic PWV or augmentation index of the arterial waveform, and the effects on the arteries lasted for at least 2 h; augmentation indices and pulse pressure amplification were linearly associated with smoking intensity (urinary cotinine levels) [29,30]. Activation of the sympathetic nervous system due to smoking increases heart rate; a higher heart rate is associated with a lower augmentation index and larger pulse pressure amplification [30]. Smoking cessation reduced augmentation indices and pulse wave velocity in the aorta, which decreased aortic blood pressure [31].

The strengths of the present study lie in the recruitment of real-world participants free of severe cardiovascular comorbidities and drugs able to influence aortic elastic properties. As far as we know, our study shows for the first time using ROC curve analysis gender differences related to the vascular effects of smoking, demonstrating a lower SPY cutoff value for increased arterial stiffness in female compared to male smokers in two groups of male and female smokers, respectively, matched for age, PWV, and arterial age. SPY more severely impaired arterial age in male compared to female smokers, probably related to the cardiovascular protective anti-atherogenic effects of estrogens in young and middle-aged women included in the present study. Brachial and aortic augmentation indices were significantly higher in female smokers only after adjusting for heart rate, probably related to the higher heart rates recorded in this group. The findings of the present study have clinical implications, considering the prognostic importance of increased arterial stiffness for cardiovascular risk.

Cardiovascular screening using an Arteriograph may be beneficial in smokers. Monitoring of brachial augmentation index and pulse wave velocity and early detection of increased arterial stiffness are especially important in female smokers, in order to reclassify patients to higher or lower risk groups and avoid severe cardiovascular events. The Arteriograph provides a simple method to assess PWV, augmentation indices, and arterial age in daily clinical practice, similar to the conventional measurement of blood pressure. The present study encourages lifestyle changes in smokers, emphasizing the importance of smoking cessation—especially in women—for positive vascular outcomes in order to reduce the burden of cardiovascular disease. Smokers are aware of the harmful effect of smoking, but they continue due to the addiction and the belief of improved stress management, but the beneficial effect on stress reduction was questioned by a recent study [21].

Study limitations: The most important limitations of the present study are the cross-sectional study design, the observational nature of the study, the small patient number, self-reported data about smoking habits, the use of arterial stiffness and arterial age as surrogate markers of cardiovascular risk, and missing data related to lipid profile, fasting blood glucose, and serum creatinine. The cross-sectional study design does not provide data about a cause–effect relationship between the variables. Aortic PWV measurement provided by the Arteriograph has also been questioned, but it has been previously validated against invasive methods [32]. There exists a trend toward underestimation in self-reported data considering that smoking is a socially undesirable behavior, but any misclassification might have been independent of smoking status [2,30]. The present study was conducted in Caucasian subjects, and the results cannot be extended to other populations. Despite higher blood pressure values in male smokers, no significant differences were found for PWV, AA, or AixAo between male and female smokers. The cut-off value of 9.7 m/s used in the present study for increased PWV—despite European Guidelines recommending a threshold of 10 m/s for pathological values [33]—should emphasize the prophylactic character of the present study. The statistical inference based on logistic regression and ROC curve analysis was acceptable, since it is well known that sample sizes of 70–100 are enough for the construction of confidence regions and making significance tests. We do not expect large changes in the results by increasing the sample size, but further research in this direction will be of interest. We can also consider this issue as a practical study limitation, or an open problem, since even 10 times higher sample size will not guarantee not to be trapped into ignorance regions [34].

Further larger follow up studies are needed in male and female smokers to confirm the findings of the present study and to compare the collagen content of the arterial wall considering gender.

## 5. Conclusions

SPY is significantly associated with an increased arterial stiffness, regardless of gender and with an increased systolic blood pressure in the aorta in female smokers. The present study shows gender differences for arterial stiffness and arterial age in smokers. A lower exposure to cigarette smoke is needed to increase arterial stiffness in female compared to male smokers.

## Figures and Tables

**Table 1:** Characteristics, results, and gender differences of the study population considering Wilcoxon test and ANOVA.

Variables	Male Smokers (Means ± SD) (*n* = 71)	Female Smokers (Means ± SD) (*n* = 76)	Wilcoxon Test (*p*)	ANOVA (*p*)
Age (years)	38 ± 17	36 ± 13	2816 (0.64)	0.96 (0.32)
HR (beats/minute)	68.04 ± 9.48	75.25 ± 11.60	1758 (0.0002)	16.86 (0.00006)
SPY (pack year)	12.36 ± 13.15	6.43 ± 8.81	3503 (0.001)	10.44 (0.001)
Daily cigarette consumption	17 ± 9	11 ± 6	3716 (<0.01)	20.91 (<0.01)
Smoking period (years)	12.49 ± 9.79	9.18 ± 7.45	3265 (0.02)	5.35 (0.02)
Body mass index (kg/m^2^)	27.2 ± 5.29	24.55 ± 7.11	3594 (0.0005)	6.49 (0.01)
Systolic Blood Pressure (mmHg)	129 ± 17	119 ± 13	3827 (<0.01)	19.10 (<0.01)
Diastolic Blood Pressure (mmHg)	77 ± 12	67 ± 8	4146 (<0.01)	34.95 (<0.01)
PWV (m/s)	9.47 ± 6.69 m/s	8.48 ± 2.26	2825 (0.62)	1.47 (0.22)
Arterial age (years)	42.19 ± 16.17	40.19 ± 18.21	2886 (0.46)	0.49 (0.48)
AixBrach (%)	−43.77 ± 25.85	−34.27 ± 30.79	2195.5 (0.05)	4.07 (0.05)
AixBrach 75% (%)	−47.62 ± 24.94	−33.79 ± 28.54	1816 (0.0006)	9.73 (0.002)
SBPAo (mmHg)	119 ± 17	112 ± 15	3485 (0.002)	7.16 (0.008)
AixAo (%)	15.44 ± 13.09	17.81 ± 24.22	2227.5 (0.06)	0.53 (0.47)
AixAo 75% (%)	11.56 ± 12.72	18.88 ± 22.36	1515 (<0.01)	5.83 (0.02)

SD = standard deviation, standard deviations were calculated from the unbiased estimator of the variance; SPY = smoking pack years, PWV = pulse wave velocity, HR = heart rate, AixBrach = brachial augmentation index, AixBrach 75% = brachial augmentation index corrected for a heart rate of 75 beats/minute, AixAo = aortic augmentation index, AixAo 75% = aortic augmentation index corrected for a heart rate of 75 beats/minute, SBPAo = systolic blood pressure in the aorta, *p* < 0.01 was considered significant for Wilcoxon test.

**Table 2:** Correlations between SPY and arteriography variables in male and female smokers.

Correlations of SPY with	Correlations of SPY in Male Smokers (r, *p*)	Correlations of SPY in Female Smokers (r, *p*)	Probability
AixBrach	0.51 (<0.001)	0.06 (0.59)	0.001
SBPAo	0.24 (0.03)	0.26 (0.02)	0.44
AixAo	0.51 (<0.01)	−0.28 (0.01)	<0.01
PWV	0.11 (0.35)	0.51 (<0.01)	0.003
AA	0.68 (<0.01)	0.35 (0.001)	0.003

SPY = smoking pack years, AixBrach = brachial augmentation index, SBPAo = systolic blood pressure in the aorta, AixAo = aortic augmentation index, PWV = pulse wave velocity, AA = arterial age.

**Table 3:** Results of binary logistic regression analysis with SPY as an independent variable.

Sex	Outcome	*p*	Hosmer–Lemershow Goodness of Fit	Beta	OR	95% CI	Nagelkerke R-Square
Male	AixBrach ˃ 10%	<0.01	0.558 ˃ 0.5	0.09	1.09	1.021–1.173	0.284
	PWV ˃ 9.7 m/s	<0.01	0.324 < 0.5	0.14	1.15	1.078–1.244	0.562
Female	AixBrach ˃ 10%	<0.01	0.02 < 0.5	0.01	1.01	0.951–1.090	0.882
	PWV ˃ 9.7 m/s	<0.01	0.040 < 0.5	0.32	1.37	1.13–1.66	0.470

SPY = smoking pack years, Nagelkerke R-square = predicted variance of the outcome, AixBrach = brachial augmentation index, PWV = pulse wave velocity.

**Table 4:** Results of receiver-operating characteristic (ROC) curve analysis.

Gender of Smokers	Test Variable	State Variable	AUC (95%CI)	*p*	Cut-Off Value for SPY	Sensitivity	Specificity
Female smokers	SPY	AixBrach ˃ 10%	0.8 (0.702–0.898)	0.04	7.5	82.1%	66.7%
	SPY	SBPAo ˃ 130 mmHg	0.856 (0.763–0.949)	<0.01	9	87.7%	63.6%
	SPY	PWV ˃ 9.7 m/s	0.886 (0.805–0.967)	<0.01	7.5	92.9%	70%
Male smokers	SPY	AixBrach ˃ 10%	0.858 (0.695–1)	0.017	18.5	74.6%	75%
	SPY	PWV ˃ 9.7 m/s	0.93 (0.871–0.989)	0.01	18.5	93%	85.7%

AixBrach = brachial augmentation index, PWV = pulse wave velocity, SBPAo = systolic blood pressure in the aorta, SPY = smoking pack years.

## References

[1] Sandru C. (2012). Smoking prevalence in Romania. A Secondary Data Analysis. Bull. Transilv. Univ. Brasov Ser. VII Soc. Sci. Law.

[2] Gorber S.C., Schofield-Hurwitz S., Hardt J., Levasseur G., Tremblay M. (2009). The accuracy of self-reported smoking: A systematic review of the relationship between self-reported and cotinine-assessed smoking status. Nicotine Tob. Res..

[3] Mozos I., Gligor S. (2015). Blood pressure variables, arterial stiffness, endothelial function and arterial age in sedentary and physically active smokers. Jokull.

[4] Gaenzer H., Neumayr G., Marschang P., Sturm W., Kirchmair R., Patsch J.R. (2001). Flow-mediated vasodilation of the femoral and brachial artery induced by exercise in healthy nonsmoking and smoking men. J. Am. Coll. Cardiol..

[5] Hassan Talukder M.A., Johnson W.M., Varadharaj S., Lian J., Kearns P.N., El-Mahdy M.A., Liu X., Zweier J.L. (2011). Chronic cigarette smoking causes hypertension, increased oxidative stress, impaired NO bioavailability, endothelial dysfunction, and cardiac remodeling in mice. Am. J. Physiol. Heart Circ. Physiol..

[6] Miyata S., Noda A., Ito Y., Iizuka R., Shimokata K. (2015). Smoking acutely impaired endothelial function in healthy college students. Acta Cardiol..

[7] Mozos I. (2014). The link between ventricular repolarization variables and arterial function. J. Electrocardiol..

[8] Van Bortel L. (2016). Arterial stiffness: From surrogate marker to therapeutic target. Artery Res..

[9] Vlachopoulos C., Xaplanteris P., Aboyans V., Brodmann M., Cífková R., Cosentino F., De Carlo M., Gallino A., Landmesser U., Laurent S. (2015). The role of vascular biomarkers for primary and secondary prevention. A position paper from the European Society of Cardiology Working Group on peripheral circulation: Endorsed by the Association for Research into Arterial Structure and Physiology (ARTERY) Society. Atherosclerosis.

[10] Lee H.Y., Oh B.H. (2010). Aging and arterial stiffness. Circul. J..

[11] Paulista Markus M.R., Stritzke J., Baumeister S.E., Siewert U., Baulmann J., Hannemann A., Schipf S., Meisinger C., Dörr M., Felix S.B. (2013). Effects of smoking on arterial distensibility, central aortic pressure and left ventricular mass. Int. J. Cardiol..

[12] Townsend R.R., Wilkinson I.B., Schiffrin E.L., Avolio A.P., Chirinos J.A., Cockcroft J.R., Heffernan K.S., Lakatta E.G., McEniery C.M., Mitchell G.F. (2015). Recommendations for improving and standardizing vasular research on arterial stiffness. A scientific statement from the American Heart Association. Hypertension.

[13] Regnauld V., Thomas F., Safar M.E., Osborne-Pellegrin M., Khalil R.A., Pannier B., Lacolley P. (2012). Sex difference in cardiovascular risk. Role of pulse pressure amplification. J. Am. Coll. Cardiol..

[14] Yang T., Barnett R., Jiang S., Yu L., Xian H., Ying J., Zheng W. (2016). Gender balance and its impact on male and female smoking rates in Chinese cities. Soc. Sci. Med..

[15] Weinberger A.H., Platt J.M., Shuter J, Goodwin R.D. (2016). Gender differences in self-reported withdrawal symptoms and reducing or quitting smoking three years later: A prospective, longitudinal examination of US adults. Drug Alcohol. Depend..

[16] Mozos I., Filimon L., Gligor S. (2014). Body mass index, blood pressure and arteriography variables in a middle and aged population. HealthMed.

[17] Laurent S., Cockroft J., Van Bortel L., Boutouyrie P., Giannattasio C., Hayoz D., Pannier B., Vlachopoulos C., Wilkinson I., Struijker-Boudier H. (2006). Expert consensus document on arterial stiffness: Methodological issues and clinical applications. Eur. Heart J..

[18] Prignot J. (1987). Quantification and chemical markers of tobacco-exposure. Eur. J. Respir. Dis..

[19] Wood D.M., Mould M.G., Ong S.B.Y., Baker E.H. (2005). “Pack year” smoking histories: What about patients who use loose tobacco?. Tob. Control.

[20] Leone A., Landini L., Leone A. (2010). What is tobacco smoke? Sociocultural dimensions of the association with cardiovascular risk.. Curr. Pharm. Des..

[21] Jeon M., Jeong H., Lee K., Yim J. (2014). The acute effect of smoking a single cigarette on vascular status, SpO2, and stress level. Med. Sci. Monit..

[22] Raghuveer G., White D.A., Hayman L.L., Woo J.G., Villafane J., Celermajer D., Ward K.D., de Ferranti S.D., Zachariah J. (2016). Cardiovascular consequences of childhood secondhand tobacco smoke exposure: Prevailing evidence, burden, and racial and socioeconomic disparities: A systematic statement from the American Heart Association. Circulation.

[23] Faarvang A.S., Rordam Preil S.A., Nielsen P.S., Beck H.C., Kristensen L.P., Rasmussen L.M. (2016). Smoking is associated with lower amounts of arterial type I collagen and decorin. Atherosclerosis.

[24] Seet R.C.S., Loke W.M., Khoo C.M., Chew S.E., Chong W.L., Quek A.M., Lim E.C., Halliwell B. (2012). Acute effects of cigarette smoking on insulin resistance and arterial stiffness in young adults. Atherosclerosis.

[25] Singh R., Sharma S., Singh R.K., Mahdi A.A., Singh R.K., Gierke C.L., Cornelissen G. (2016). Effect of gender, age, diet and smoking status on chronomics of circulating plasma lipid components in healthy Indians. Clin. Chim. Acta.

[26] Wang J.W., Hu D.Y. (2015). Association of serum cotinine levels and the parameters of vascular structure and function in never-smoking adults. J. Am. Soc. Hypertens..

[27] Mahmud A., Feely J. (2003). Effect of smoking on arterial stiffness and pulse pressure amplification. Hypertension.

[28] Vlachopoulos C., Aznaouridis K., Bratsas A., Ioakeimidis N., Dima I., Xaplanteris P., Stefanadis C., Tousoulis D. (2015). Arterial stiffening and systemic endothelial activation induced by smoking. The role of COX-1 and COX-2. Int. J. Cardiol..

[29] Vlachopoulos C., Alexopoulos N., Panagiotakos D., O’rourke M.F., Stefanadis C. (2004). Cigar smoking has an acute detrimental effect on arterial stiffness. Am. J. Hypertens..

[30] Tabara Y., Takahashi Y., Setoh K., Muro S., Kawaguchi T., Terao C., Kosugi S., Sekine A., Yamada R., Mishima M. (2013). Increased aortic wave reflection and smaller pulse pressure amplification in smokers and passive smokers confirmed by urinary cotinine levels: The Nagahama Study. Int. J. Cardiol..

[31] Jatoi N.A., Jerrard-Dunne P., Feely J., Mahmud A. (2007). Impact of smoking and smoking cessation on arterial stiffness and aortic wave reflection in hypertension. Hypertension.

[32] Tarnoki A.D., Tarnoki D.L., Bogl L.H., Medda E., Fagnani C., Nisticò L., Stazi M.A., Brescianini S., Lucatelli P., Boatta E. (2013). Association of body mass index with arterial stiffness and blood pressure components: A twin study. Atherosclerosis.

[33] Van Bortel L.M., Laurent S., Boutouyrie P., Chowienczyk P., Cruickshank J.K., De Backer T., Filipovsky J., Huybrechts S., Mattace-Raso F.U., Protogerou A.D. (2012). Expert consensus document on the measurement of aortic stiffness in daily practice using carotid-femoral pulse wave velocity. J. Hypertens..

[34] Vansteelandt S., Goetghebeur E. (2001). Analyzing the sensitivity of generalized linear models to incomplete outcomes via the IDE algorithm. J. Comput. Graph. Stat..

